# P-864. Systematic Review and Meta-analysis of the Impact of Infectious Diseases Consultation on Management and Outcomes of *Staphylococcus aureus* Bacteremia in Children

**DOI:** 10.1093/ofid/ofae631.1055

**Published:** 2025-01-29

**Authors:** Haripriya Santhanam, Nirmal Muthukumarasamy, Mariana Kim Hsieh, Karen Brust, Melanie Wellington, Toshio Naito, Riley J Samuelson, Alexandre Marra, Takaaki Kobayashi

**Affiliations:** Eastern Iowa Health Center, Iowa City, Iowa; University of Iowa Hospital and clinic, Sioux Falls, South Dakota; University of Iowa Hospitals and Clinics, Iowa City, Iowa; University of Iowa Hospitals & Clinics, Iowa City, Iowa; University of Iowa, Iowa City, Iowa; Juntendo University Faculty of Medicine, Bunkyo, Tokyo, Japan; University of Iowa Libraries, Iowa City, Iowa; University of Iowa Hospital and Clinics, iowa city, Iowa; University of Iowa Hospitals and Clinics, Iowa City, Iowa

## Abstract

**Background:**

Staphylococcus aureus is a common cause of both community-acquired and nosocomial bacteremia in children. Multiple studies evaluating the role of infectious disease consultation (IDC) in adult patients with Staphylococcus aureus bacteremia (SAB) have shown a protective effect of IDC on mortality and recurrence rates. However, there is limited data available regarding the impact of IDC on outcomes of SAB in the pediatric population.

Literature Search on the Impact of Infectious Disease Consultation in Pediatric Patients with Staphylococcus aureus Bacteremia

**Figure:**
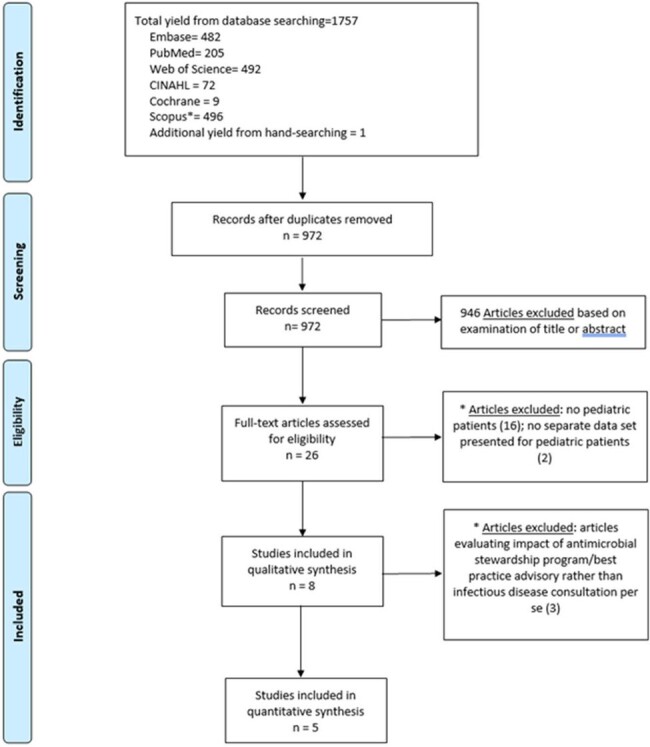

**Methods:**

This systematic literature review and meta-analysis were performed per the Preferred Reporting Items for Systematic Reviews and Meta-Analysis (PRISMA) statement and the Meta-Analysis of Observational Studies in Epidemiology (MOOSE) guidelines. A search strategy to identify publications about SAB and IDC in children was developed in collaboration with a health sciences librarian. The primary outcomes were all-cause mortality and SAB recurrence rates. Crude or unadjusted numbers were used for the pooled odds ratios (ORs) as adjusted ORs were not available in all articles.

Meta-analysis of outcomes of Staphylococcus aureus Bacteremia in pediatric patients

**Figure:**
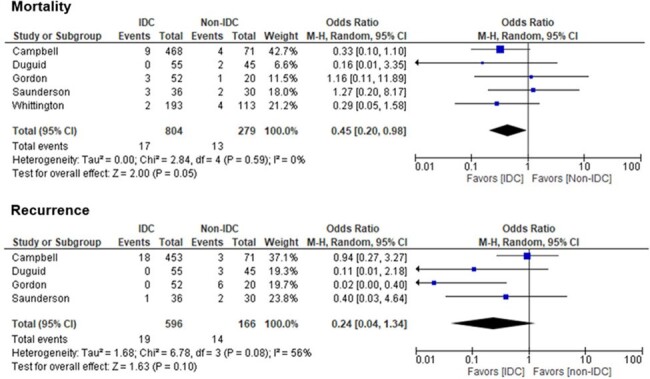

**Results:**

Among 972 articles screened, 8 studies were included in the systematic review, of which, 2 were retrospective cohort studies, 2 were prospective cross-sectional studies, and 4 were quasi-experimental studies. The quality of 6 studies was considered good ( >18 of 28 possible points) per the Downs and Black quality tool while two studies were considered fair (15 – 18 points). Five of 8 studies directly evaluated the impact of IDC on outcomes in pediatric SAB and included in the meta-analysis. Pooled results showed IDC was associated with significantly lower mortality in pediatric SAB with low heterogeneity (pooled OR = 0.44, 95% confidence interval [CI]: 0.20−0.97, I2 = 0%). IDC was associated with lower recurrence rates, however this was not statistically significant with moderate heterogeneity (pooled OR = 0.24, 95% CI: 0.04 to 1.34, I2 = 56%).

Funnel Plot of Mortality and Recurrence in Pediatric Patients with Staphylococcus aureus Bacteremia

**Figure:**
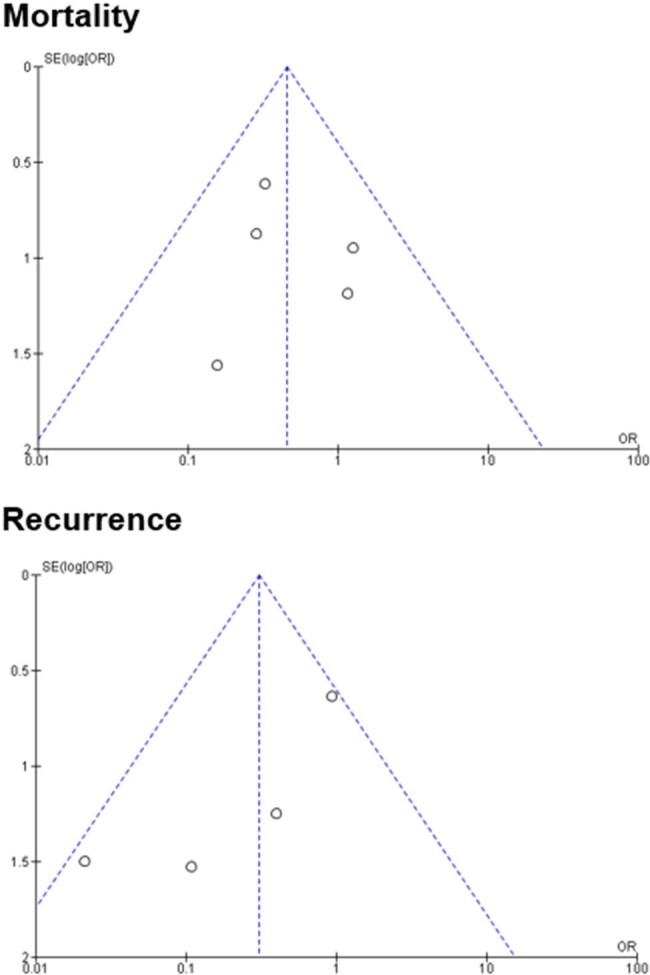

**Conclusion:**

Our study suggests that IDC significantly improves the mortality of pediatric patients with SAB. Given there have been only five papers evaluating the same topic, with most being retrospective studies at a single center, a multicenter prospective study will be required. Our study provides a strong argument in favor of policies such as “automatic” IDC for children with SAB.

**Disclosures:**

**All Authors**: No reported disclosures

